# Integrating multiple networks for protein function prediction

**DOI:** 10.1186/1752-0509-9-S1-S3

**Published:** 2015-01-21

**Authors:** Guoxian Yu, Hailong Zhu, Carlotta Domeniconi, Maozu Guo

**Affiliations:** 1 College of Computer and Information Sciences, Southwest University, Chongqing, China; 2 Department of Computer Science, Hong Kong Baptist University, Hong Kong; 3 Department of Computer Science, George Mason University, VA, US; 4 School of Computer Science and Technology, Harbin Institute of Technology, Harbin, China

## Abstract

**Background:**

High throughput techniques produce multiple functional association networks. Integrating these networks can enhance the accuracy of protein function prediction. Many algorithms have been introduced to generate a composite network, which is obtained as a weighted sum of individual networks. The weight assigned to an individual network reflects its benefit towards the protein functional annotation inference. A classifier is then trained on the composite network for predicting protein functions. However, since these techniques model the optimization of the composite network and the prediction tasks as separate objectives, the resulting composite network is not necessarily optimal for the follow-up protein function prediction.

**Results:**

We address this issue by modeling the optimization of the composite network and the prediction problems within a unified objective function. In particular, we use a kernel target alignment technique and the loss function of a network based classifier to jointly adjust the weights assigned to the individual networks. We show that the proposed method, called MNet, can achieve a performance that is superior (with respect to different evaluation criteria) to related techniques using the multiple networks of four example species (yeast, human, mouse, and fly) annotated with thousands (or hundreds) of GO terms.

**Conclusion:**

MNet can effectively integrate multiple networks for protein function prediction and is robust to the input parameters. Supplementary data is available at https://sites.google.com/site/guoxian85/home/mnet. The Matlab code of MNet is available upon request.

## Introduction

Determining the functional roles of proteins is important to understand life at molecular level and has great biomedical and pharmaceutical implications [1-3]. Proteins with similar amino acids often have similar functions. Additionally, functions are often performed by proteins physically interacting with each other, or located in the same complex [4], or having similar structures. The availability of a large variety of genomic and proteomic data makes it possible to predict protein functions *in silico *by leveraging these data. More accurate functional inference of proteins can be achieved by integrating these heterogeneous sources of genomic and proteomic data [5,6]. The competitive algorithms from the first large-scale community-based critical assessment of protein function annotation (CAFA) also took advantage of heterogeneous data sources [2,7-9]. Some data integration based hybrid methods do something more sophisticated, i.e., incorporating the evolution knowledge [8], the pathways information [10] and negative examples [11,12].

A number of computational methods have been suggested to integrate heterogeneous data for inferring protein (or gene) functions [6,13]. Most of these techniques follow the same basic paradigm: first, they generate various functional association networks (one or more networks for one data source) that encode the implicit information of shared functions of proteins in each data source. Then these *individual *networks (or kernels) are combined, through a weighted sum, into a *composite *network, where the weights are optimized using labels, each label corresponding to a distinct protein function. Next, the composite network, along with the function labels, are given in input to a network (or kernel) based classification algorithm [5,14-16] to compute the likelihood of a specific function label for a protein.

The functional association network is an inherent and widely applied representation for encoding information of shared protein functions from high-throughput proteomic (or genomic) data sources (i.e., protein-protein interactions (PPI), protein sequences). In this representation, a node in the network corresponds to a protein, and the weights of the edges of connected nodes are specified to capture the evidence (or reliability) of shared functions derived from one data source. These weights are computed by a specific similarity metric for a given data source. For example, string kernels [17] for protein sequences, Pearson's correlation coefficients for gene expression profiles. In this way, each data source can be transformed into a network (or kernel). To leverage the networks derived from heterogeneous data sources to predict protein functions, some approaches first train individual classifiers on these networks and then use ensemble learning techniques to combine these classifiers [7,9,11,18]. Another set of algorithms first integrate these networks into a composite network and then train network-based learning methods [5,14-16]. In this study, we focus on the second kind of algorithms.

Current techniques on integrating multiple networks can be mainly divided into two categories: (i) several approaches model the composite kernel optimization and the final predictor training as separate problems. As such they may not necessarily result in optimal predictors [15,16]. (ii) Some methods optimize the composite network and the predictor for each functional label separately [5,14]. Since protein functions are inter-correlated and most functional labels often have a relatively small number of member proteins, these algorithms ignore the interrelationship among labels, which can often be used to boost the prediction accuracy [3,19]. Furthermore, they have to resort to time consuming special techniques (i.e., parameter tuning, regularization) to avoid the over-fitting problem and to optimize a composite network for each label.

To overcome the limitations of existing techniques, we introduce a new approach to integrate *M*ultiple *Net*works (MNet) for prediction of protein functions. Unlike the aforementioned methods, MNet jointly optimizes the multiple network integration and the network-based classifier for a set of function labels in a unified objective function. In addition, MNet takes into account the unbalanced label problem in protein function prediction, and incorporates a label weighted scheme into the unified objective function to give more emphasis to the functional labels with fewer proteins. Our empirical study on four publicly available species (yeast, human, fly, and mouse, with different number of individual networks), annotated with thousands of GO terms, shows that MNet performs better (according to different evaluation criteria) than other related techniques. Furthermore, MNet, unlike the competitive methods, enables an easy selection of suitable parameters.

## Related work

Different proteomic data sources (i.e., protein sequences, PPI networks and protein domains) often capture proteins' properties in different aspects, and have different correlations with different GO terms [1,20]. Yang *et al*. [21] and Teng *et al*. [22] observed that the GO term similarities have different correlations with different proteomic data sources. Therefore, integrating these data sources can often enable a more comprehensive view of proteins and their functions. Recently, several studies have observed a significant improvement in protein function prediction when multiple heterogenous biological data sources are integrated. To name a few, Pavlidis *et al*. [23] integrated heterogeneous data sources in three different ways: (i) *early *integration concatenates all feature vectors from different data sources of a protein into a single feature vector; (ii) *intermediate *integration computes the functional association network for each data set separately and then combines them; (iii) *late *integration trains a support vector machine (SVM) on each network (or kernel) and then combines the resulting discriminant values. Their study revealed that different data sources have different qualities, and setting different weights for different networks can enhance the accuracy of protein function prediction. Lanckriet *et al*. [5] proposed a semi-definite programming based SVM method to get the optimal weights on individual networks. Tsuda *et al*. [14] constructed an optimal combination of weights on individual networks using convex optimization. Mostafavi *et al*. [15] determined the optimal function-specific composite network by solving a linear regression problem. These methods constructed a composite network for each functional label. Since there are often more than hundreds of functional labels, and these labels are highly unbalanced and inter-correlated, these algorithms are often confronted with the over-fitting problem and require massive computational resources.

More recently, some researchers advocated for the computation of optimal weights on individual networks for a group of labels, and achieved better performance than the methods operating on single labels. Mostafavi *et al*. [16] introduced a method, called *SW*, that simultaneously optimizes the weights on individual networks with respect to a group of related functional labels by solving a single-constrained linear regression problem. The optimal weights maximize a form of kernel-target alignment [24] between the composite network and the target network, which is defined based on the functional relationships implied by the functions of proteins. However, merely maximizing the kernel target alignment does not necessarily result in an optimal composite network for the network-based classifier. Yu *et al*. [25] proposed a method, called ProMK, that combines the composite network optimization with respect to a group of functions and the network based classifier in a unified objective function. ProMK can selectively integrate multiple networks and can construct an optimal composite network directly targeted to the network based classification, but it suffers from the parameter selection problem, and does not take into account the intrinsic unbalanced label problem in protein function prediction.

In this study, we build a composite network optimized for a linear neighborhood propagation classifier. The resulting method is called MNet. MNet iteratively optimizes the weights assigned to the individual networks and the loss of the classifier according to a unified objective function. We show that the unified objective function can boost the accuracy of protein function prediction according to several evaluation criteria. Furthermore, MNet is more robust than other related approaches for a wide range of parameter values.

MNet has a close relationship with multiple kernel learning, which is a popular topic in machine learning [26], and it's also widely applied in biological data mining [5,14,25]. Wang *et al*. [27] introduced a method called Optimal Multiple Graphs learning (OMG) to integrate multiple graphs into a composite one for graph-based semi-supervised learning. Shiga *et al*. [28] proposed a method called LIG. LIG first partitions each individual graph into several locally informative subgraphs via soft spectral clustering and then integrates these subgraphs into a composite one for graph-based classification. A protein can have several different functions and these functions are inter-correlated, thus protein function prediction from multiple data sources can also be transformed into a multi-label multiple kernel learning problem [3,25]. Multi-label multiple kernel learning methods often learn a composite kernel for each binary label and thus have a complexity linear to the number of labels. Bucak et al. [29] suggested a method called multiple kernel learning by stochastic approximation, whose complexity is sub-linear to the number of labels.

MNet is different from the aforementioned approaches to integrating multiple networks in several ways. ProMK, OMG, and LIG assign weights to the individual networks solely based on their smoothness loss: the smaller the value of the smoothness loss for a network, the larger the weight assigned to this network. However, our empirical study in this paper shows that, a smaller value of the smoothness loss on an individual network does not necessarily imply that the network is a better predictor. In contrast, MNet assigns weights to the individual networks not only based on the smoothness loss, but also on the kernel-target alignment. Therefore, MNet alleviates the drawback of the existing methods. Furthermore, MNet constructs a composite network that is coherent to all the labels, whereas most multiple kernel learning algorithms optimize a composite kernel for each binary label, or optimize the composite kernel and the classifier in two separative objectives.

## Method

### Network-based prediction algorithm

Let $W^{m}\in {\mathbb{R}}^{n\times n}\left(m\in \left\{1,2,\ldots ,M\right\}\right)$ be a weight matrix corresponding to the *m*-th individual functional association network. Each node of a network corresponds to one of the *n *proteins, and the entry $W_{i,j}^{m}\geq 0$ is the association (similarity, or reliability of interaction) between proteins *i *and *j *in the *m*-th data source. Among the *n *proteins, the first *l *proteins have confirmed annotation, and the functional annotation of the remaining *u *= *n *- *l *proteins needs to be predicted. These annotated proteins have *C *distinct functions, and each annotated protein has a subset of the *C *functions. Each of these *C *functions corresponds to a Gene Ontology (GO) term in one of the three sub-branches (Biological Process, Molecular Function, Cellular Component) of the GO [30]. The functions of the *i*-th protein is represented as a label vector **y***_i _*∈ {0|1}*^C^*, where *y_ic _*= 1 if the *i*-th protein is confirmed to have the *c*-th function, otherwise, *y_ic _*= 0. For an unlabeled protein *j*, *y_jc _*= 0 (*l *<*j *≤ *n*, 1 ≤ *c *≤ *C*). Here, we denote the predicted likelihood vector of the *i*-th protein as **f***_i _*∈ **R***^C ^*, and *f_ic _*represents the likelihood that the *i*-th protein has the *c*-th function.

Let $W=\sum_{m=1}^{M}\alpha_{m}W^{m}$ be the association matrix of the composite network, where *α*_*m *_≥ 0 is the weight assigned to the *m*-th network, representing its relevance towards the prediction task. Many network-based algorithms [13] extend the guilt-by-association rule [31], or exploit the cluster structure of protein complex [4] to predict protein functions using the PPI networks. Inspired by these work, we use a linear neighborhood propagation algorithm [32] on the composite network *W *to predict protein functions:

(1) $$\begin{array}{c} f=\text{arg }{\text{min}}_{f}\overset{l}{\underset{i=1}{\sum }}||f_{i}-y_{i}|{|}_{2}^{2}+\overset{n}{\underset{i=1}{\sum }}||f_{i}-\underset{j\in N\left(i\right)}{\sum }W_{ij}f_{j}|{|}_{2}^{2}\\ s.t.\overset{n}{\underset{j=1}{\sum }}W_{ij}=1 \end{array}$$

where *N*(*i*) is the set of proteins that have connections with the *i*-th protein, 0 ≤ *W*_*ij *_≤ 1 is the (*i, j*)-th entry of *W *, and *I *is an *n *× *n *identity matrix. The first term in Eq. (1) enforces the prediction to be close to the initial annotation of the *l *proteins, and it is often viewed as the *empirical *loss term. The minimization of the second term enforces that the functions assigned to an unlabeled protein *j *are determined by the functions of its connected proteins in *W*; as such the second term acts as a *smoothness *loss term [33]. Eq. (1) is motivated by the observation that interacting proteins are more likely to share similar functions [31], and two proteins with similar amino acids often have similar functions [17]. The above equation can be rewritten in matrix notation as:

(2) $$F=\text{arg }{\text{min}}_{F}tr\left({\left(F-Y\right)}^{T}H\left(F-Y\right)\right)+tr\left(F^{T}LF\right)$$

Here, *Y *= [**y**_1_, **y**_2_,⋯,**y**_*n*_], *F *= [**f**_1_, **f**_2_,⋯,**f**_*n*_] ∈ **R**^*n *× *C*^, *H *is an *n *× *n *diagonal matrix with *H*_*ii *_= 1 if *i *≤ *l*, and *H*_*ii *_= 0 otherwise, *L *= (*I - W *)*^T ^*(*I - W*) and *I *is an *n *× *n *identity matrix, *tr*(·) and *T *are the matrix trace and transpose operators, respectively. By taking the differentiation of Eq. (2) to *F *and setting the differentiation to zero, *F *can be computed as:

(3) $$F={\left(H+L\right)}^{-1}HY$$

The functional labels are organized in a hierarchy (a directed acyclic graph for GO terms, and a tree for MIPS FunCat). The more specific the functional label is in the hierarchy, the fewer member proteins this label has. If a protein has a confirmed functional label, this protein is also annotated with its ancestor labels in the hierarchy [3,30,34]. Therefore, protein function prediction is an unbalanced classification problem and to achieve a good prediction it's important to take into account this issue [3,11]. Eq. (1) ignores the unbalanced problem and considers all functional labels as equal. To address this limitation, we modify *y_ic _*into ${\tilde{y}}_{ic}=y_{ic}\text{log}\frac{N}{n_{c}^{+}}$ ($N=\sum_{c=1}^{C}n_{c}^{+},n_{c}^{+}$ proteins annotated with the *c*-th function). The added factor has the effect of putting more emphasis on functional labels that are more specific. This forces the optimizer to focus on the more specific functions, versus the general ones which have more member proteins. We set $\tilde{Y}=\left[{\tilde{\text{y}}}_{1},\cdots ,{\tilde{\text{y}}}_{n}\right]$, and $F={\left(H+L\right)}^{-1}H\tilde{Y}$.

### Kernel target alignment

Given $W_{ij}=\sum_{m=1}^{M}\alpha_{m}W_{ij}^{m}$ and the available functional association networks ${\left\{W^{m}\right\}}_{m=1}^{M}$, the accuracy of protein function prediction is determined by ***α ***= [*α*_1_, *α*_2_,⋯,*α*_*M*_]. [24] and [35] have shown that the target aligned kernel (network) can boost the performance of kernel-based classification and regression. To compute the weights to be assigned to the *M *individual networks, we resort to a form of kernel-target alignment algorithm [24] as follows:

(4) $$\begin{array}{c} \alpha =\text{arg}{\text{min}}_{\alpha }tr\left({\left(K-W\right)}^{T}\left(K-W\right)\right)\\ s.t.W=\overset{M}{\underset{m=1}{\sum }}\alpha_{m}W^{m},\alpha_{m}\geq 0 \end{array}$$

where $K\in {\mathbb{R}}^{n\times n}$ is the induced target network of functional labels, defined as $K=\sum_{c=1}^{C}K^{c}$, where *K^c ^*is the *c*-th induced target network computed as:

$$K^{c}\left(i,j\right)=\left\{\begin{array}{cc} {\left(\frac{n_{c}^{-}}{l}\right)}^{2} & \text{if}y_{ic}=y_{jc}=1\\ \frac{n_{c}^{+}n_{c}^{-}}{l^{2}} & \text{if}y_{ic}y_{jc}=0\&y_{ic}+y_{jc}=1\&i,j\leq 1\\ 0, & \text{otherwise} \end{array}\right)$$

where $n_{c}^{-}$ is the number of proteins which are not annotated with the *c*-th function. Since a functional label often has a relatively small number of member proteins $n_{c}^{+}<n_{c}^{-}$ and ${\left(\frac{n_{c}^{-}}{l}\right)}^{2}>\frac{n_{c}^{+}n_{c}^{-}}{l^{2}}$. From the definition, the more functions two proteins have in commom, the larger the entry (corresponding to the weight of the edge between them) in the target network is. This idea was adapted to define the target network [15,16] and to reconstruct the functional association network [36]. Mostafavi *et al*. [15,16] set the entry (corresponding to the edge between two proteins such that one has the *c*-th function and the other doesn't) in the target network as $-\frac{n_{c}^{+}n_{c}^{-}}{n^{2}}$. In contrast, we set the entry as $\frac{n_{c}^{+}n_{c}^{-}}{l^{2}}$. The reason is that the available GO term annotation of proteins is incomplete, is updated regularly and suffer from a large research bias [3,21,37]. As such, *y*_ic _= 0 should not be simply interpreted as if the *i*-th protein does not have the *c*-th function. Furthermore, for a to be predicted protein *j*, if *W*(*i*; *j*) is large, from the guilty by association rule, protein j is likely to share some functions with the *i*-th protein. By minimizing Eq. (4), we aim at crediting larger weights to the networks which consider highly similar proteins which share *more *functions, and smaller weights to networks which consider highly similar proteins which share *fewer *or no functions. By doing so, we can assign larger weights to networks that are coherent with functional labels.

Based on the fact that *tr*(*KW*) = *vec*(*K*)*^T ^vec*(*W*), where *vec*(*K*) is the vectorization operator that stacks the columns of *K *on top of each other, we can rewrite Eq. (4) as a non-negative linear regression problem:

(5) $$\begin{array}{c} \alpha =\text{arg}{\text{min}}_{\alpha }tr\left({\left(V_{K}-V_{W}\alpha \right)}^{T}\left(V_{K}-V_{W}\alpha \right)\right)\\ s.t.\;\alpha_{m}\geq 0,1\leq m\leq M \end{array}$$

where *V_K _*= *vec*(*K*), $V_{W}=\left[vec\left(W^{1}\right),\cdots ,vec\left(W^{M}\right)\right]\in {\mathbb{R}}^{\left(n\times n\right)\times M}$.

### The unified objective function

Mostafavi *et al*. [15,16] first utilized Eq. (4) to determine ***α ***for the individual networks, and then applied a Gaussian Random Field classifier [33] on the composite network *W *to predict protein functions. However, the composite network *W *optimized from the target network *K *is not necessarily optimal for the classifier, since the optimization of the composite network and of the classifier is divided into two separate objectives. To avoid this limitation, we attempt to integrate these two objectives into a unified function as follows:

(6) $$\begin{array}{c} \alpha =\text{arg}{\text{min}}_{\alpha }tr({\left(V_{K}-\overset{M}{\underset{m=1}{\sum }}\alpha_{m}v_{W}^{m}\right)}^{T}\left(\left(V_{K}-\overset{M}{\underset{m=1}{\sum }}\alpha_{m}v_{W}^{m}\right)\right)\\ +\lambda tr\left(F^{T}{\left(I-\overset{M}{\underset{m=1}{\sum }}\alpha_{m}W^{m}\right)}^{T}\left(I-\overset{M}{\underset{m=1}{\sum }}\alpha_{m}W^{m}\right)F\right)\\ +\lambda tr\left({\left(F-\tilde{Y}\right)}^{T}H\left(F-\tilde{Y}\right)\right)\\ s.t.\;\alpha_{m}\geq 0,1\leq m\leq M \end{array}$$

where $v_{W}^{m}$ is the *m*-th column vector of *V_W_*. Eq. (6) combines the objectives of network-based classification and of target network alignment. Therefore, we can enforce the composite network to be coherently optimal with respect to both objectives.

The above objective function is convex with respect to *F *and *α*, respectively. Here, we introduce an EM [38] style algorithm to iteratively optimize *F *(or *α*) while keeping *α *(or *F*) constant. For a fixed *α*, we can obtain *F *directly from Eq. (2). For a fixed *F*, using *tr*(*α_m_W*) = *α_m_tr*(*W*) and *tr*(*K - W*) = *tr*(*K*) - *tr*(*W*), Eq. (7) can be rewritten as:

(7) $$\begin{array}{c} \alpha =\text{arg}{\text{min}}_{\alpha }-2\alpha^{T}V_{W}^{T}V_{K}+\alpha^{T}V_{W}^{T}V_{K}\alpha \\ +\lambda \left(-2\alpha^{T}\mu +\alpha^{T}\Theta \alpha \right)\\ s.t.\;\alpha_{m}\geq 0,1\leq m\leq M \end{array}$$

where Θ is an *M *× *M *matrix with $\Theta \left(m_{1},m_{2}\right)=tr\left(F^{T}{\left(W^{m_{1}}\right)}^{T}W^{m_{2}}F\right)$, ***μ ***is an *M *× 1 vector with *μ*_*m *_= *tr*(*F^T^W^m^F*). The other terms in Eq. (6) are constant for a fixed *F *and irrelevant for ***α***, thus they are not included in Eq. (7). Taking the partial derivatives with respect to ***α***, we can obtain the following solution:

(8) $$\alpha ={\left(V_{W}^{T}V_{W}+\lambda \Theta \right)}^{-1}\left(V_{W}^{T}V_{K}+\lambda \mu \right)$$

It is easy to see that, if *λ *= 0, only the kernel target alignment criterion is used to optimize ***α***, and MNet is similar to SW.

The MNet algorithm is presented in Algorithm 1. *F*^*t *^and ***α***^*t *^are the computed values for *F *and ***α ***at the *t*-th iteration, *maxIter *and *θ *are the maximum number of iterations and the convergence threshold, respectively.

**Algorithm 1 **MNet: Integrating Multiple Networks for Protein Function Prediction

Input:

   ${\left\{W_{m}\right\}}_{m=1}^{M}$ functional association networks, $\tilde{Y}$, *λ*, *maxIter*, *θ*

Output:

Predicted likelihood score vectors ${\left\{f_{j}\right\}}_{j=l+1}^{n}$

1: Initialize $\alpha_{m}^{1}=1\left(1\leq m\leq M\right)$, and *t *= 1

2: **while ***t *<*maxIter *and |*δ*| >*θ ***do**

3:   *t *= *t *+ 1

4:   Compute *F^t ^*using Eq. (3)

5:   Compute ***α***^*t *^using Eq. (8)

6: *δ *= |***α***^*t *^- ***α***^*t*-1^|

7: **end while**

### Complexity analysis

Several components in Eq. (8) (i.e., $V_{W}^{T}V_{W}\in R^{M\times M}$, $V_{W}^{T}V_{K}\in R^{M\times 1}$ and ${\left(W^{m_{1}}\right)}^{T}W^{m_{2}}\in R^{n\times n}$) can be computed before the iterative process. The time complexity of *tr*(*F^T^WF *) is *O*(*Cn*^2^). Θ is an *M *× *M *symmetric matrix, in each iteration there are *M *(*M *+ 1)/2 elements to be computed, so the time complexity of computing Θ is *O*(*M *(*M *+ 1) × *Cn*^2^). The complexity of matrix inverse in Eq. (3) is *O*(*n*^3^). To avoid large matrix inverse, iterative approximation algorithms (i.e. Conjugate Gradient) can be applied. Since the computation complexity of matrix inverse in Eq. (8), and the complexity of ***μ ***is smaller than Θ, the overall time complexity of MNet is *max*{*O*(*M*^2^*TCn*^2^)*, O*(*Tn*^3^)}. *T *is the number of iterations for convergency. In practice, *T *is no more than 10. In our study, the association matrices of the individual networks and the composite network are all sparse with *O*(*n*) non-zero elements. For this reason, the time complexity of the above operations can be greatly reduced. The main difference between MNet and ProMK is that ProMK just uses ***μ***, so its time complexity is *max*{*O*(*MTCn*^2^)*, O*(*Tn*^3^)}. Given that the number of individual networks is much smaller than the number of proteins and functional labels, MNet has similar complexity with ProMK.

## Results and discussion

### Yeast, human, mouse and fly datasets

We evaluate our methodology on benchmark networks of four datasets obtained from the study by [16], which cover four species: yeast, human, mouse, and fly. The Yeast dataset includes 44 functional association networks, the Human dataset includes 8 networks, the Mouse dataset consists of 10 networks, and the Fly dataset has 38 networks. These datasets are publicly available at http://morrislab.med.utoronto.ca/~sara/SW/, and more information about them can be found in [16].

We annotated the proteins in each dataset using the recently updated GO term annotation (access date: 2014-05-13) in three sub-ontologies, namely biological process (BP) functions, molecular functions (MF), and cellular component (CC) functions, respectively. Each protein is also annotated with its ancestral function labels. As suggested by Pandey *et al*. [19], the functional labels which have too few member proteins are not likely to be testable in the wet lab, and thus of no interest to biologists. We retained the function labels which have at least 10 member proteins. In addition, we removed the functional labels that have more than 300 member proteins: these functional labels are too general and their prediction is not as critical as for the others [25,39]. The statistic of these datasets is given in Table 1. In the table, the BP labels are the biological process functions (or terms), the MF labels are the molecular functions, and the CC labels are the cellular component functions in the Gene Ontology.

**Table 1:** Dataset statistics.

Dataset	#Proteins	#Networks	#BPs	#MFs	#CCs
Yeast	3904	44	1089	307	224
Human	13281	8	3413	681	438
Mouse	21603	10	4123	818	511
Fly	13562	38	1883	481	315

'#Proteins' represents the number of proteins in a dataset, '#Networks' means the number of functional association networks, '#BPs' denotes the number of BP labels, '#MFs' denotes the number of MF labels, and '#CCs' denotes the number of CC labels.

### Comparing algorithms and evaluation metrics

We compared our proposed MNet with other related algorithms: ProMK [25], SW [16], OMG [27], LIG [28], and MSkNN [7]. MSkNN first trains a weighted majority vote [31] classifier (similar to a weighted *k*NN) on each individual network, and then integrates these classifiers for protein function prediction; it achieves competent performance on the first large-scale community based critical assessment of protein function annotation [2]. The details of the other comparing methods were introduced in the section of Related Work, and their parameter setting is discussed in the Additional File 1.

The quality of protein function prediction can be evaluated according to different criteria, and the choice of evaluation metrics differentially affects different prediction algorithms [2]. For a fair and comprehensive comparison, five evaluation metrics are used in this paper, namely *MacroF1*, *MicroF1*, *Fmax*, function-wise Area Under the Curve (*fAUC *), and protein-wise AUC (*pAUC *). These evaluation metrics are extensively applied to evaluate the performance of multilabel learning algorithms and protein function prediction [2,7,25,40]. More information about these evaluation metrics is provided in the Additional File 1. For an evaluation metric, since there are more than hundreds (or thousands) of labels for a dataset, a small performance difference between two comparing algorithms is also significant.

### Protein function prediction

**Figure 1:**
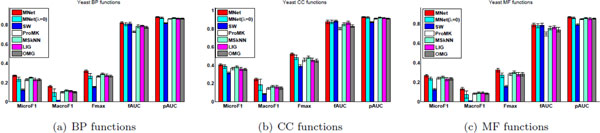
**Prediction of the Biological Process (BP) functions, the Cellular Component (CC) functions, and the Molecule functions (MF) of the *Yeast *dataset**. The groups from left to right give the prediction results with respect to the evaluation metrics *MicroF1*, *MacroF1*, *Fmax*, *fAUC*, and *pAUC *for the different algorithms.

**Figure 2:**
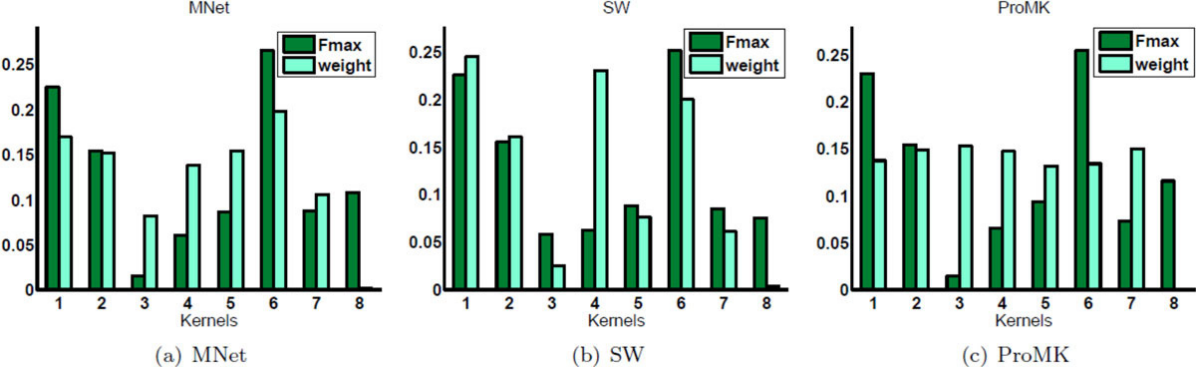
**Network relevance estimation using MNet, SW, and ProMK on the *Human *dataset annotated with *BP *functions**. For each group of bars, the left one shows the *Fmax *value on the individual network, and the right one gives the weight assigned to the same network.

**Figure 3:**
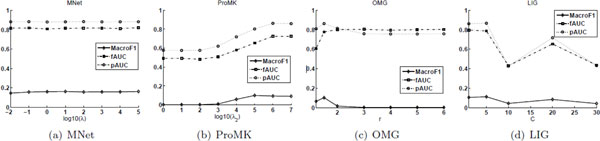
**Parameter sensitivity of MNet, ProMK, OMG, and LIG on the *Yeast *dataset annotated with *BP *functions**.

We use five-fold cross validation to investigate the performance of the algorithms in predicting protein function. More specifically, we divide each dataset into five disjoint folds. In each round, we take four folds as the training data and the remaining fold as the testing set, in which the proteins are considered as unlabeled and to be predicted. We record the results on the testing data to measure the performance. The parameters of the comparing methods are optimized via five-fold cross validation on the training data. Figure 1 gives the prediction performance of the comparing methods on the BP, CC, and MF functions of Yeast, respectively. The results on the other datasets are reported in Figures 1-3 of the Additional File 1.

From the figures, we have several important observations. MNet almost always performs better than the other algorithms (including MS*k*NN) across all the evaluation metrics and all the three sub-ontologies (BP, CC, and MF) of GO, and the performance of the other methods fluctuate with respect to the different evaluation metrics. MNet also often outperforms MNet(*λ *= 0), which first uses kernel target alignment to obtain the composite network, and then applies classification on the composite network to predict protein functions. The difference between MNet and MNet(*λ *= 0) shows that it is important and beneficial to unify the composite network optimization with the prediction task on the composite network. MNet(*λ *= 0) performs better than SW in most cases, and both of them are solely based on the kernel target alignment to compute the weights on individual networks. The reason is that MNet (*λ *= 0) sets the weight of the edge between two proteins (such that one has the *c*-th function and the other currently does't) as $\frac{n_{c}^{+}n_{c}^{-}}{l^{2}}$, whereas SW sets it as $-\frac{n_{c}^{+}n_{c}^{-}}{n^{2}}$. For the evaluation metric *fAUC*, SW and MNet sometimes have comparable results, but SW often loses to MNet in other evaluation metrics. The reason is three-fold: (i) SW optimizes the composite network using kernel target alignment in advance, and then it performs binary classification on the composite network, whereas MNet unifies the optimization of the composite network and the network-based classifier for all the labels; (ii) SW specifies the label bias (often negative, since each label is annotated with a small number of proteins) for each binary label and MNet also sets the label bias (inversely proportional to the number of member proteins) to each binary label; (iii) *fAUC *is a function-centric evaluation metric and it equally averages the AUC scores of different labels, and the other evaluation metrics (i.e., *Fmax *and *pAUC *) do not favor the binary predictor. In fact, most functional labels are only annotated with a rather small number of proteins. For this reason, we observe that the true positive rate is close to 1 in a wide range of false positive rates for a large number of functional labels. This fact also accounts for similar *fAUC *results of MNet and SW.

Another observation is that SW often loses to other comparing methods on *MacroF1 *and *MicroF1*. There are two reasons for this behavior: (i) SW applies binary classification on the composite network, but the other comparing algorithms do network-based classification for all the labels; (ii) *MicroF1 *and *MacroF1 *are computed based on the transformed binary indicative label vectors, and the binary indicative vector is derived from the largest elements of **f***_i _*for each protein (see the metric definition in the Additional File 1 for more information); the other three metrics do not apply the binary transformation of **f**_*i*_. MS*k*NN uses a classifier ensemble to integrate multiple networks, and sometimes gets comparable results to other algorithms, which take advantage of the composite network to fuse multiple networks. These results show that classifier ensembles are another effective way to fuse multiple data sources for protein function prediction.

ProMK and OMG also integrate the optimization of composite network and the classifier, but they only use the loss of the classifier on the individual networks to determine the weights. LIG first utilizes soft spectral clustering to partition each input individual network into several subnetworks, and then determines the weights of these subnetworks solely based on the loss of the classifier on them. SW constructs a composite network in advance, and then train a classifier on the composite network to predict protein functions. Since it optimizes the composite network and the classifier on the composite network into two separate objectives, it often loses to other comparing algorithms. These facts support our motivation to unifying the composite network construction based on kernel target alignment and the network-based predictor optimization.

Each dataset has more than thousands (or hundreds) of labels. These labels are highly unbalanced and each protein is annotated with a very small number of labels (i.e., each protein in the Human dataset on average has 13.52 BP labels and there are a total of 3413 BP labels). Since *MacroF1 *is more driven by the labels associated to fewer proteins, and *MicroF1 *is more affected by the labels associated to a larger number of proteins, the algorithms have larger values of *MicroF1 *than *MacroF1*. The difference between MNet and the other algorithms (including SW, which also considers the problem of unbalanced labels) on *MacroF1 *is more obvious than that on *MicroF1*. This observation indicates that MNet can handle the unbalanced problem much better than the other methods.

#### The Benefit of Weighting Functional Labels

Some researchers [3,11,39] suggested that protein function prediction should be addressed as an unbalanced classification algorithm. Additional experiments were conducted to investigate the benefit of using $\tilde{Y}$ (weighted) in place of *Y *(unweighted). $\tilde{Y}$ differentially weights the labels, and puts more emphasis on the labels that have fewer member proteins. In contrast, *Y *equally weights all the labels. The definition of *Y *and $\tilde{Y}$ are provided in the section of Method. We report the results of MNet using $\tilde{Y}$ (weighted) and *Y *(unweighted) in Table 2 of the Additional File 1.

**Table 2:** Runtime (in seconds).

Dataset	GO	MNet	SW	ProMK	MSkNN	LIG	OMG
Yeast	BP	2256.26	151.88	72.61	16.60	938.10	65.51
	CC	282.10	36.39	31.84	12.47	272.89	15.76
	MF	390.10	46.07	36.83	12.42	322.11	18.97

Human	BP	19923.15	120.09	628.30	42.15	10309.56	447.01
	CC	1003.46	17.57	350.92	31.69	1496.33	96.61
	MF	1633.55	25.42	369.92	32.62	2195.25	116.59

MNet based on $\tilde{Y}$ performs better than MNet based on *Y *, especially for the BP labels, which are more unbalanced than the CC and the MF labels. *MacroF1 *is more affected by the labels that contains fewer proteins, and the performance difference between MNet based on $\tilde{Y}$ and MNet based on *Y *is more obvious for *MacroF1 *than for the other metrics. This fact shows that MNet based on $\tilde{Y}$ can more accurately predict the labels with few member proteins than MNet based on *Y *, and explicitly considering the unbalanced problem in data integration based protein function prediction can boost the prediction accuracy. These results support our motivation to use $\tilde{Y}$ instead of *Y*. However, we point out that there is still room to handle the unbalanced label problem for protein function prediction more efficiently, and how to achieve a more efficient weighting scheme for the labels is an important future direction to pursue.

### Network relevance estimation

Different networks present different levels of quality for protein function prediction. To investigate whether MNet can assign a large weight to a network that can produce accurate predictions, and assign a small weight to a network that poorly predicts protein functions, we recorded the results of MNet (see Eq. (1)) for individual networks and the corresponding weights (*α_m_*). We also recorded the results and the weights of SW and ProMK on individual networks. For a fair comparison and a better visualization, we scale these weights in the interval [0, 1] as follows: $\alpha_{m}/\sum_{i=1}^{M}\alpha_{m}$.

Figure 2 gives the *Fmax *values on the eight individual networks of the Human dataset (annotated with the BP labels), and the optimized weights on these networks. The corresponding results with respect to *MacroF1 *and *fAUC *are reported in Figures 3 and 4 of the Additional File 1. We also provide the results on the Human dataset (annotated with the CC and the MF labels) in the Additional File 1 (see Figures 5 and 6).

From the Figure, we can observe that all three algorithms achieve the largest *Fmax *value on the 6-th network, and the *Fmax *value on each individual network has a similar rank among the eight individual networks across the different methods, i.e., the *Fmax *value on the 1st network ranks second according to MNet, SW, and ProMK. MNet assigns a larger weight on the 6-th network as compared to the weights for the other networks. In contrast, neither SW nor ProMK assigns the largest weight to the 6-th network. MNet, SW, and ProMK give the smallest weight to the 8-th network, though these methods do not produce the lowest *Fmax *for the 8-th network. The reason is that the 8-th network produces rather large smoothness loss values as compared to those of the other networks. Since *λ*_2 _is given a large value, ProMK assigns nearly equal weights to the first 7 networks. Because the smoothness loss value on the 8-th network is much larger than for the others, ProMK assigns zero weight to the 8-th network. Note that for small *λ*_2 _values, ProMK can only use one network and produces deteriorated results (see our parameter analysis in the next subsection). The *Fmax *values on the first three networks progressively decrease, and the weights assigned by MNet and SW to these networks also decrease. In contrast, the weights assigned by ProMK do not follow this trend. ProMK assigns larger weights to the 2nd and 3rd networks. The *Fmax *values on the next three (4-th, 5-th, and 6-th) networks, as well as the weights assigned by MNet, progressively increase, but the weight assigned by SW to the 4-th network is larger than those assigned to the 5-th and 6-th networks, and the weights assigned by ProMK progressively decrease. All these three methods give a smaller *Fmax *value to the 7-th network than to the 6-th; both MNet and SW assign a smaller weight to the 7-th network than to the 6-th, but ProMK assigns a larger weight to the 7-th network than to the 6-th. ProMK, OMG and LIG use only the smoothness loss to assign weights to the individual networks. The smaller the value of the smoothness loss for a network is, the larger the weight assigned to it is. The value of the smoothness loss of ProMK on the 3rd network is smaller than the values of the other networks, thus ProMK assigns a weight to the 3rd network that is larger than the ones assigned to other networks. However, the value of *Fmax *of this network is the lowest. This conflictual scenario shows that assigning a weight to a network merely based on the smoothness loss is not always reasonable. This justifies our motivation to unifying the kernel target alignment with the loss of classifier in one objective function, and also provides evidence as for why MNet works better than the other algorithms. These observations also apply to the results provided in the Additional File 1.

Another interesting observation for Figure 6 in the Additional File 1 is that MNet, SW, and ProMK give the highest *Fmax *value to the 1st network of the Human dataset (annotated with MF functions), instead of to the 6-th network. In the Human dataset, the 1st network is derived from protein domain composition and the 6-th is a PPI network. This observation supports the statement that different data sources have different correlation with the GO terms. Lan *et al*. [7] also observed that the prediction of MF functions using sequence similarity is more accurate than that based on PPI information, and the prediction of BP functions based on PPI networks is more reliable than that based on sequence similarity. Regardless of this difference for the proteins of Human annotated with MF functions, MNet shows similar trends for the weight and the *Fmax *values assigned to the individual networks. In contrast, neither SW nor ProMK manifests such behavior.

If we take the *Fmax *value of an individual network as its quality, we can conclude that MNet can assign weights to the individual networks that are proportional to their quality, whereas SW and ProMK cannot. This observation also helps us understand why MNet achieves a performance that is better than that of SW and ProMK.

### Parameter sensitivity

Some of the algorithms used for comparison need to tune several parameters, and the specification of these parameters affect the performance. These parameters and their suggested ranges are listed in Table 1 of the Additional File 1. The result of MNet depends on *λ*, whose purpose is to balance the kernel target alignment and the loss of the classifier on the composite network. ProMK relies on the specification of *λ*_2 _to determine the weights on individual networks. OMG needs to tune the power size *r *on the weights and LIG requires to set the number of subnetworks for each input network. To study the parameter sensitive of these algorithms, for MNet, we vary *λ *in {10^-2^, 10^-1^,⋯,10^5^}; for ProMK, we vary *λ*_2 _in {10^0^, 10^1^,⋯,10^7^}; for OMG, we vary *r *in {1.2, 1.5, 2, 3, 4, 5, 6}, and for LIG, we vary *C *in {1, 5, 10, 20, 30}. For each setting value of the parameter, we execute five-fold cross validation as in the previous experiment, and report the average result. The results of these methods on *Yeast *(annotated with BP functions) under different values of the parameters are reported in Figure 3. We also provide similar results (*Yeast *annotated with CC and BP functions, and *Human *annotated with BP functions) in Figures 7-9 of the Additional File 1.

When *λ *is set to a small value (i.e., *λ *= 10^-2^), a small emphasis is put on the classification task and a large stress on the kernel target alignment; as such, the results of MNet slightly deteriorate. These results also support our statement that optimizing the kernel target alignment (or composite network) does not necessarily result in the optimal composite network for classification. When *λ *= 1 or above, MNet has a stable performance and outperforms the other methods. This trend also justifies our motivation to unifying the kernel target alignment and the classifier on the composite network in a combined objective function.

When *λ*_2 _is small, only one network can be chosen by ProMK, and therefore ProMK achieves a relatively poor result in this case. When *λ*_2 _increases to a value larger than 103, more kernels are used to construct the composite network, and the results of ProMK progressively improve and achieve stability when most of the networks are used to construct the composite one. The best setting of *r *for OMG is *r *= 1.5; for larger values, the results worsen and they become stable when *r *≤ 3. As for LIG, the values *C *= 1 and *C *= 5 often give the best results, and LIG's performance sometimes fluctuates sharply for other settings of *C*.

From these results, we can draw the conclusion that MNet can select an effective *λ*'s value from a wide range, and MNet is less affected by the parameter selection problem than ProMK and the other competitive algorithms.

### Runtime analysis

We also recorded the running times of MNet and the other comparing methods on the Yeast and Human datasets. The results are given in Table 2. All the methods are implemented in Matlab (R2011a 64-bit). The specification of the experiment platform is: CentOS 5.6, Intel Xeon X5650 and 32GB RAM.

From Table 2, we can observe that MNet often takes more time than the other methods. As the number of functional labels reduces, the runtime cost of MNet decreases sharply. The reason is that MNet has to compute the trace norm not only for individual networks, but also for the pairwise networks (see Eq. (7)). In contrast, ProMK, OMG, and LIG only compute the trace norm for individual networks. The running time of MNet is often no more than *M *(the number of individual networks) times the cost of ProMK, which is consistent with our previous complexity analysis. MS*k*NN does not learn weights on individual networks; as such it always runs faster than the other methods. SW first applies kernel target alignment to fuse multiple networks into a composite one, and then predicts protein functions using the composite network; it often ranks second (from fastest to lowest) among the comparing methods. Both ProMK and OMG iteratively optimize the weights on individual networks; they have similar runtime costs and lose to SW and MS*k*NN. LIG takes more time than the other methods; sometimes it is also slower than MNet. The reason is that LIG applies time-consuming eigen-decomposition for soft spectral clustering to divide each individual network into several subnetworks, and then combines these subnetworks into a composite one for function prediction. Given the superior effectiveness of MNet, it is desirable to use MNet to integrate multiple networks for protein function prediction. However, seeking efficient and effective ways to utilize multiple networks for function prediction remains an important research direction to explore.

## Conclusions

In this paper, we study how to integrate multiple functional networks for accurate protein function prediction and propose a method called MNet. MNet unifies the optimization of a composite network and the optimization of a predictor on the composite network in a single objective. An extensive empirical study shows that MNet can predict protein functions more accurately than related competitive methods, and it's also less affected by the parameter selection issue.

Protein functions are rather difficult to predict, i.e., proteins are often multifunctional and promiscuous, and the functional annotations of proteins are incomplete and error prone [2,3,21]. There are many avenues for future improvements for protein function prediction. For example, incorporating pathway information, evolutional knowledge, and reducing the noisy in the individual networks before the integration. In addition, an input network can have some high quality subnetworks and low quality subnetworks; it is promising to design algorithms to discover and differentiate these subnetworks, and to integrate the high quality subnetworks to enhance the prediction accuracy, and to discard the low quality ones (or assign very small weights to these subnetworks) to reduce their destructive effects.

## Competing interests

The authors declare that they have no competing interests.

## Authors' contributions

**GY **and **CD **designed the algorithm and drafted the manuscript, **GY **performed the experiments, **MG **participated in result analysis and revised the manuscript, **HZ **conceived the program and finalized the manuscript. All the authors read and approved the final manuscript.

## Supplementary Material

Additional file 1 **Supplementary file of 'Integrating multiple networks for protein function prediction'**. Click here for file

## Declarations

Publication costs for this article was funded by the corresponding author.

This article has been published as part of *BMC Systems Biology *Volume 9 Supplement 1, 2015: Selected articles from the Thirteenth Asia Pacific Bioinformatics Conference (APBC 2015): Systems Biology. The full contents of the supplement are available online at http://www.biomedcentral.com/bmcsystbiol/supplements/9/S1
