# Supplementary material for: Integrating multiple networks for protein function prediction
Source: BMC Syst Biol. 2015 Jan 21;9(Suppl 1):S3. doi: 10.1186/1752-0509-9-S1-S3 (PMC4331678; doi:10.1186/1752-0509-9-S1-S3)
Supplement: Additional file 1 — Supplementary file of 'Integrating multiple networks for protein function prediction'. [file 1752-0509-9-S1-S3-S1.pdf]

Supplementary file of 'Integrating multiple networks for protein function prediction'

Guoxian Yu[1,2,*], Hailong Zhu[2,*], Carlotta Domeniconi[3] and Maozu Guo[4]
[1]College of Computer and Information Sciences, Southwest University, Chongqing, China
[2]Department of Computer Science, Hong Kong Baptist University, Hong Kong
[3]Department of Computer Science, George Mason University, VA, USA
[4]School of Computer Science and Technology, Harbin Institute of Technology, Harbin, China
[*]Contact: guoxian85@gmail.com, hlzhu@comp.hkbu.edu.hk

November 21, 2014

Parameter Setting of Comparing Algorithms

Some algorithms depend on the tuning of parameters. We reported the parameter tuning ranges of these algorithms in Table 1. For MNet, we found λ in the specified range can get rather stable performance. Given that, we set $\lambda = 1$ for the experiments on all the datasets. ProMK [1] requires to tune λ_1 and λ_2, given the weight α_m mainly depends on λ_2, we simply set $\lambda_1 = 1$ and optimize λ_2 in the specified range. OMG [2] needs to specify λ_1 and r, similar to ProMK, we set $\lambda_1 = 1$ and tuned r in the range specified in the third row of Table 1. LIG [3] needs to specify several parameters, we used the default parameter settings provided by the authors and tuned C (the number of subgraphs for each input graph) in the specified range. We observed that LIG ($C = 5$) often produced the best performance, and it sometimes got similar results with LIG ($C = 1$). We set $C = 5$ for LIG for the experiments on all the datasets.

Evaluation Metrics

Here, we provide the definition of the five evaluation metrics *MacroF1*, *MicroF1*, *Fmax*, function-wise Area Under the Curve ($fAUC$) and protein-wise AUC ($pAUC$). These evaluation metrics are extensively applied to evaluate the performance of multi-label learning algorithm and protein function prediction [1, 4, 5].

Let p_c and r_c be the precision and recall of the c-th label, computed as:

$$p_c = \frac{TP_c}{TP_c + FP_c} \quad r_c = \frac{TP_c}{TP_c + FN_c}$$

TP_c, FP_c, and FN_c are the true positive, false positive, and false negative of the c-th function label.

MacroF1 is the average of harmonic mean of precision and recall of different labels:

$$MacroF1 = \frac{1}{C}\sum_{c=1}^{C}\frac{2p_c r_c}{p_c + r_c}$$

where C is the number of labels. *MacroF1* give equal weight to each label, and it is more affected by the performance of the labels containing fewer member proteins.

MicroF1 calculates the $F1$ measure on the predictions of different labels as a whole:

$$MicroF1 = \frac{\sum_{c=1}^{C} 2p_c r_c}{\sum_{c=1}^{C} p_c + r_c}$$

MicroF1 does not give equal weights to each label. The labels having more member proteins have larger impacts on *MicroF1* than the labels having fewer member proteins. Thus, *MicroF1* is more affected by the labels having more member proteins.

Fmax is a protein centric evaluation metric used in CAFA [4], *Fmax* is an F-measure computed as:

$$Fmax = \max_t \frac{2p(t)r(t)}{p(t) + r(t)}$$

where $p(t) = \frac{1}{m(t)}\sum_{i=1}^{m(t)} p_i(t)$ is the the precision at threshold $t \in [0, 1]$, $p_i(t)$ is the precision on the i-th protein, $m(t)$ is the number of proteins on which at least one prediction was made above the threshold t, $r(t) = \frac{1}{u}\sum_{i=1}^{u} r_i(t)$ is the recall across u proteins at threshold t.

$fAUC$ first computes the AUC score for each label, it gives equal weights to each AUC and then averages these AUC scores. Each AUC score is calculated as the

Table 1 Parameter Tuning Ranges

methods	parameters	objective function	reference	suggested range
MNet	λ	$\boldsymbol{\alpha} = \frac{(V_W^T V_K + \lambda \boldsymbol{\mu})}{(V_W^T V_W + \lambda \Theta)}$	Eq. (8) in the main text	$\{10^{-2}, 10^{-1}, \cdots, 10^{5}\}$
ProMK	λ_2	$\alpha_m = \frac{\eta - \mu_m}{2\lambda_2}, \eta = \frac{(2\lambda_2 + \sum_{m=1}^{M} \mu_m)}{M}$	Eq. (1) in [1]	$\{10^{0}, 10^{1}, \cdots, 10^{7}\}$
OMG	r	$\alpha_m = \frac{(\frac{1}{\lambda \Vert F - Y \Vert_2^2 + tr(F^T L_m F)})^{\frac{1}{r-1}}}{\sum_{m=1}^{M} (\frac{1}{\lambda \Vert F - Y \Vert_2^2 + tr(F^T L_m F)})^{\frac{1}{r-1}}}$	Eq. (10) in [2]	$\{1.2, 1.5, 2, 3, 4, 5, 6\}$
LIG	C	the number of subnetworks	[3]	$\{1, 5, 10, 20, 30\}$

area under the ROC curve,which is created by plotting the fraction of true positives out of the total actual positives vs. the fraction of false positives out of the total actual negatives. It measures the overall quality of the ranking induced by the classifier, instead of the quality of a single value of the threshold in that ranking.

$pAUC$ first ranks all the labels for each test protein in the descending order of the predicted likelihoods; it then varies the number of predicted labels from 1 to the total number of labels, and computes the receiver operator curve by calculating true positive rate and false positive rate for each number of predicted labels. It finally computes the area under the curve of all labels to evaluate the prediction [6].

MacroF1 and *MicroF1* require the predicted likelihood score vector $\mathbf{f}_i$ to be a binary indicator vector. Similar to [1], we take the functions corresponding to the k largest values of $\mathbf{f}_i$ as the functions of the i-th protein, k is set to the average number of functions (round to the next integer) of all proteins.

Protein Function Prediction

In the main text, we reported the protein function prediction results on the *Yeast* dataset. The experimental results on the *Human*, *Mouse* and *Fly* datasets are provided in the Fig. 1 and Fig. 4, respectively. The results in Fig. 1 and Fig. 4 give similar conclusion as in the main text. Table 2 gives the results of MNet using $\tilde{Y}$ (weighting labels) and Y (without weighting the labels).

Networks Relevance Estimation

The extra results of network relevance estimation on the *Human* dataset annotated with BP functions, CC functions and MF functions are reported in the Fig. 4-Fig. 7. These results also demonstrate MNet can assign large weights to high quality individual networks, whereas the other two comparing methods (SW and ProMK) can not always work in the same way.

Parameter Sensitivity Analysis

In the main text, we reported the results of MNet, ProMK, OMG and LIG under different values of parameters on the *Yeast* dataset annotated with BP functions. The additional results on the *Yeast* dataset annotated with CC functions, MF functions, and the *Human* dataset annotated with BP functions are given in the Fig. 8- Fig.10. These results also support the conclusion in the main text that MNet can select effective parameters in a wide range of values, and less affected by parameters selection problem than other comparing algorithms.

Author details

References

1. Yu, G., Rangwala, H., Domeniconi, C., *et al.*: Protein function prediction by integrating multiple kernels. In: Proceedings of the 23rd International Joint Conference on Artificial Intelligence (IJCAI), pp. 1869–1875 (2013)
2. Wang, M., Hua, X.-S., Hong, R., *et al.*: Unified video annotation via multigraph learning. IEEE Transactions on Circuits and Systems for Video Technology **19**(5), 733–746 (2009)
3. Shiga, M., Mamitsuka, H.: Efficient semi-supervised learning on locally informative multiple graphs. Pattern Recognition **45**(3), 1035–1049 (2012)
4. Radivojac, P., Clark, W.T., Oron, T.R., *et al.*: A large-scale evaluation of computational protein function prediction. Nature Methods **10**(3), 221–227 (2013)
5. Zhang, M.-L., Zhou, Z.-H.: A review on multi-label learning algorithms. IEEE Transactions on Knowledge and Data Engineering **26**(8), 1819–1837 (2014)
6. Bucak, S.S., Jin, R., Jain, A.K.: Multi-label learning with incomplete class assignments. In: IEEE Conference on Computer Vision and Pattern Recognition (CVPR), pp. 2801–2808 (2011)

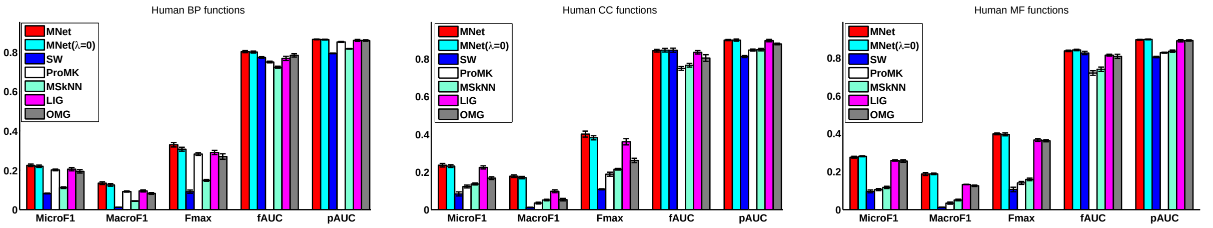

(a) BP functions (b) CC functions (c) MF functions

Figure 1 Prediction of the Biological Process (BP) functions, the Cellular Component (CC) functions, and the Molecule functions (MF) of the *Human* dataset. The groups from left to right give the prediction results with respect to the evaluation metrics *MicroF1*, *MacroF1*, *Fmax*, *fAUC*, and *pAUC* for the different algorithms.

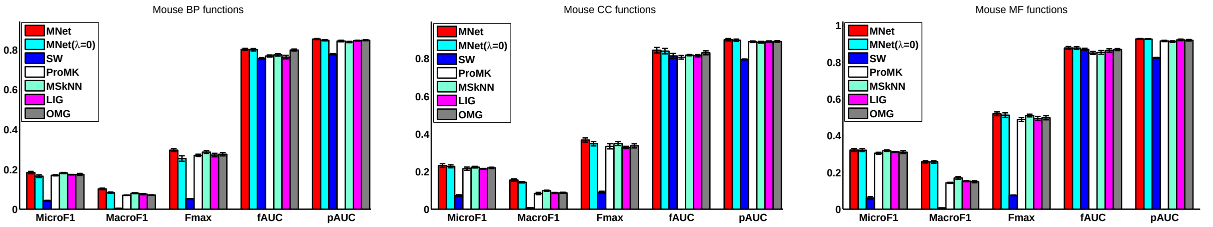

(a) BP functions (b) CC functions (c) MF functions

Figure 2 Prediction of the Biological Process (BP) functions, the Cellular Component (CC) functions, and the Molecule functions (MF) of the *Mouse* dataset. The groups from left to right give the prediction results with respect to the evaluation metrics *MicroF1*, *MacroF1*, *Fmax*, *fAUC*, and *pAUC* for the different algorithms.

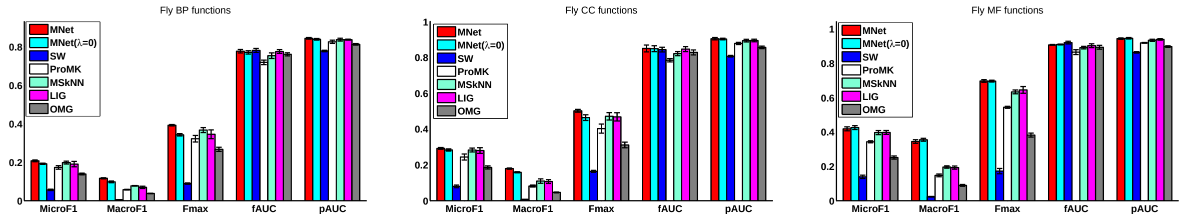

(a) BP functions (b) CC functions (c) MF functions

Figure 3 Prediction of the Biological Process (BP) functions, the Cellular Component (CC) functions, and the Molecule functions (MF) of the *Fly* dataset. The groups from left to right give the prediction results with respect to the evaluation metrics *MicroF1*, *MacroF1*, *Fmax*, *fAUC*, and *pAUC* for the different algorithms.

Table 2 MNet with and without weighting the functional labels of *Yeast*.

		MicroF1	MacroF1	Fmax	fAUC	pAUC
BP	weighted	0.2693±0.0092	0.1593±0.0091	0.3166±0.0124	0.8192±0.0144	0.8803±0.0050
	unweighted	0.2531±0.0082	0.1026±0.0072	0.2912±0.0112	0.8192±0.0114	0.8754±0.0047
CC	weighted	0.4039±0.0118	0.2431±0.0141	0.5185±0.0158	0.8743±0.0190	0.9262±0.0060
	unweighted	0.3827±0.0138	0.1437±0.0098	0.4879±0.0151	0.8749±0.0190	0.9195±0.0058
MF	weighted	0.2638±0.0144	0.1306±0.0137	0.3221±0.0188	0.7832±0.0178	0.8623±0.0079
	unweighted	0.2430±0.0131	0.0737±0.0089	0.2838±0.0161	0.7824±0.0181	0.8557±0.0080

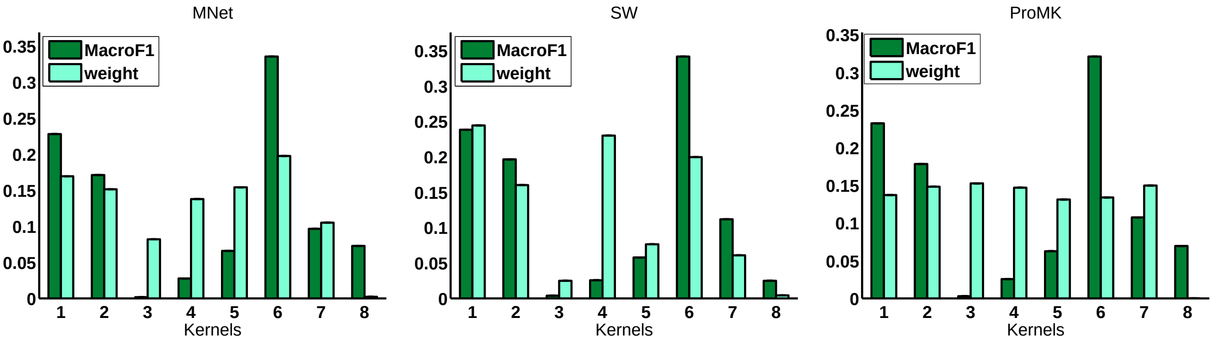

(a) MNet (b) SW (c) ProMK

Figure 4 Network relevance estimation using MNet, SW and ProMK on the *Human* dataset annotated with *BP* functions. For each group of bars, the left one shows the *MacroF1* value on the individual network, and the right one gives the weight assigned to the same network.

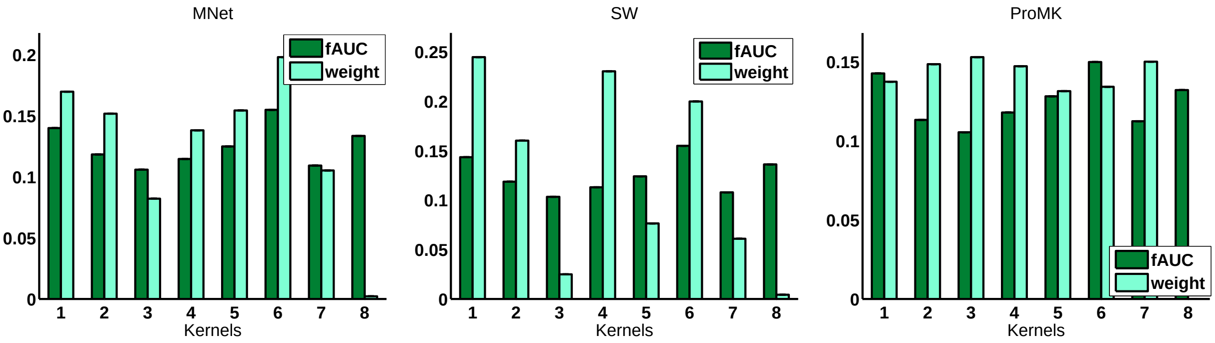

(a) MNet (b) SW (c) ProMK

Figure 5 Network relevance estimation using MNet, SW and ProMK on the *Human* dataset annotated with *BP* functions. For each group of bars, the left one shows the *fAUC* value on the individual network, and the right one gives the weight assigned to the same network.

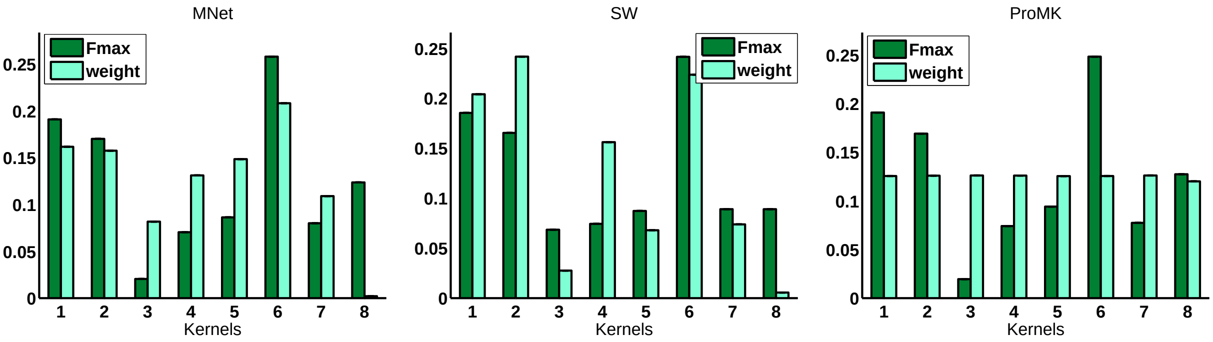

(a) MNet (b) SW (c) ProMK

Figure 6 Network relevance estimation using MNet, SW and ProMK on the *Human* dataset annotated with *CC* functions. For each group of bars, the left one shows the *Fmax* value on the individual network, and the right one gives the weight assigned to the same network.

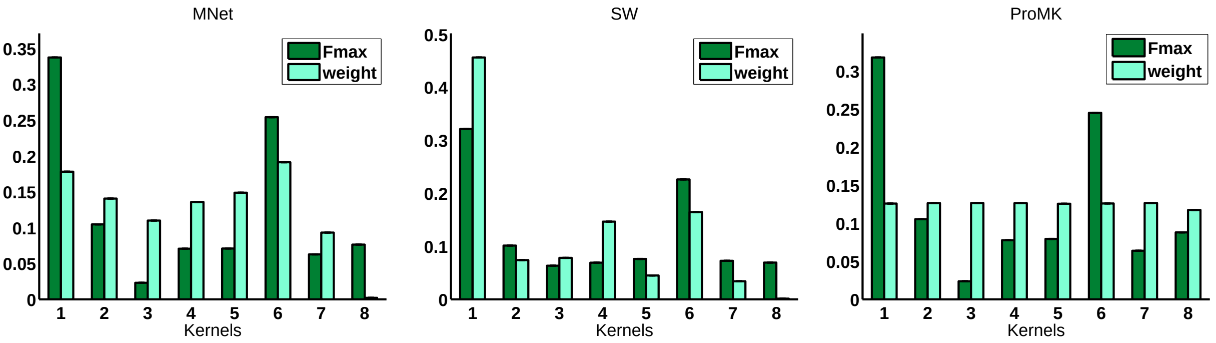

(a) MNet (b) SW (c) ProMK

Figure 7 Network relevance estimation using MNet, SW and ProMK on the *Human* dataset annotated with *MF* functions. For each group of bars, the left one shows the *Fmax* value on the individual network, and the right one gives the weight assigned to the same network.

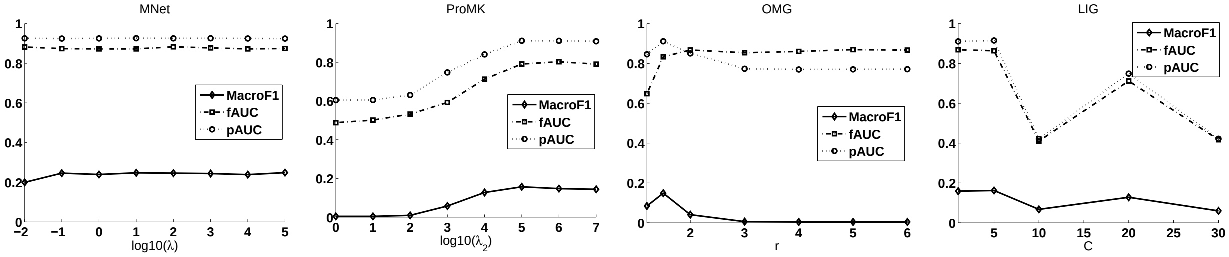

(a) MNet (b) ProMK (c) OMG (d) LIG

Figure 8 Parameter sensitive of MNet, ProMK, OMG and LIG on the *Yeast* dataset annotated with *CC* functions.

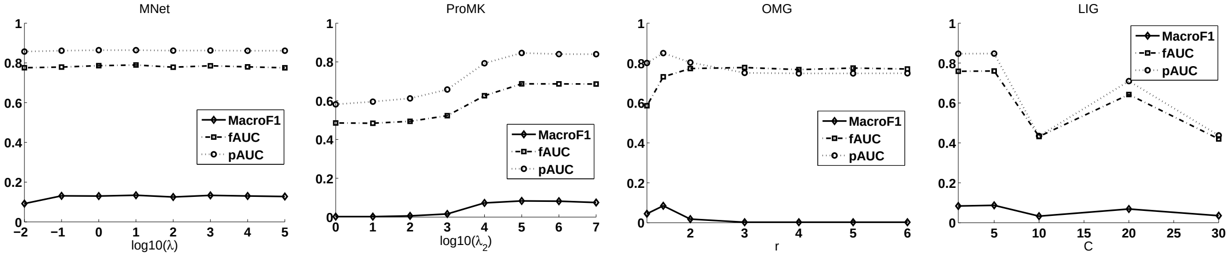

(a) MNet (b) ProMK (c) OMG (d) LIG

Figure 9 Parameter sensitive of MNet, ProMK, OMG and LIG on the *Yeast* dataset annotated with *MF* functions.

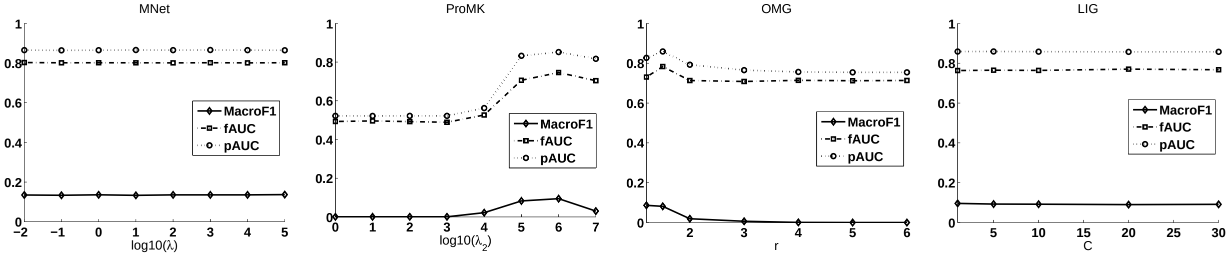

(a) MNet (b) ProMK (c) OMG (d) LIG

Figure 10 Parameter sensitive of MNet, ProMK, OMG and LIG on the *Human* dataset annotated with *BP* functions.
